# The offspring quantity–quality trade-off and human fertility variation

**DOI:** 10.1098/rstb.2015.0145

**Published:** 2016-04-19

**Authors:** David W. Lawson, Monique Borgerhoff Mulder

**Affiliations:** 1 Department of Population Health, London School of Hygiene and Tropical Medicine, London, UK; 2 Department of Anthropology, University California Davis, Davis, CA, USA

**Keywords:** life-history theory, demographic transition, parental investment

## Abstract

The idea that trade-offs between offspring quantity and quality shape reproductive behaviour has long been central to economic perspectives on fertility. It also has a parallel and richer theoretical foundation in evolutionary ecology. We review the application of the quantity–quality trade-off concept to human reproduction, emphasizing distinctions between clutch size and lifetime fertility, and the wider set of forces contributing to fertility variation in iteroparous and sexually reproducing species like our own. We then argue that in settings approximating human evolutionary history, several factors limit costly sibling competition. Consequently, while the optimization of quantity–quality trade-offs undoubtedly shaped the evolution of human physiology setting the upper limits of reproduction, we argue it plays a modest role in accounting for socio-ecological and individual variation in fertility. Only upon entering the demographic transition can fertility limitation be clearly interpreted as strategically orientated to advancing offspring quality via increased parental investment per child, with low fertility increasing descendant socio-economic success, although not reproductive success. We conclude that existing economic and evolutionary literature has often overemphasized the centrality of quantity–quality trade-offs to human fertility variation and advocate for the development of more holistic frameworks encompassing alternative life-history trade-offs and the evolved mechanisms guiding their resolution.

## 1. Introduction

The intuitive concept of a trade-off between offspring quantity and quality is central to theories of human fertility (i.e. number of births) with an independent origin in both economics and evolutionary ecology. In the conventional demographic literature, the concept is typically accredited to the economist Gary Becker. Before Becker, fertility was widely considered to be outside the realm of economic analysis [1]. In part, this followed the observation that declining fertility rates with industrialization and higher income levels appear at odds with economic rationality. Becker accounted for these trends by formalizing the notion that parents derive utility from both offspring quantity and quality, reconciling lower fertility at greater wealth as increased expenditure per child [2,3]. Since Becker, the offspring quantity–quality trade-off and related trade-offs between reproduction and female employment have dominated economic accounts of fertility [1,4]. There is also empirical support for the hypothesis that strategically reducing fertility to invest in offspring quality, particularly via formal education, is incentivized as populations undergo the demographic transition and in ‘modern’ post-demographic transition populations [5–8]. In these contexts, the socio-economic pay-offs to fertility limitation can span multiple generations. For example, Goodman *et al*. [5] demonstrate that low fertility is associated with superior descendant school performance, educational attainment, and adult income across four generations of a large Swedish cohort born during the demographic transition.

Less well known to many demographers, the quantity–quality trade-off concept has a parallel and theoretically richer origin in evolutionary ecology. Evolutionary life-history theory accounts for both species and individual-level variation in reproductive strategies in terms of the allocation of resources (time, energy, and effort) to competing functions that define the life cycle of an organism [9–11]. Since resources are finite, fundamental trade-offs must be navigated, such as that between investing in mating effort versus parental investment, in reproduction versus survival, and in offspring quantity versus quality. Life-history theory predicts that natural selection leads to the optimization of such trade-offs to maximize inclusive fitness, i.e. the production of long-term genetic descendants. This insight grounds and justifies the rational actor assumption taken *a priori* in economics in evolutionary models of organic diversity [12]. It also provides conceptual clarity by positioning fitness as the ultimate utility function guiding behaviour. This evolutionary rationale provides the unique insight that individuals are anticipated to readily forgo their own well-being, and the well-being of any individual child, provided continued reproduction maximizes fitness. Combined with the concept of bet-hedging, this rationale can explain why parents often have more children than they can seemingly afford, and introduces scepticism into economic accounts where utility is equated to individual satisfaction [13]. Economic concepts, such as ‘human capital’, have been synthesized into evolutionary models of fertility [14]. Many demographers have also embraced the complementarity of evolutionary and economic perspectives (e.g. [4,12,15]).

The objectives of this review are to: (i) overview the origin and current application of the quantity–quality trade-off concept in evolutionary ecology; and (ii) consider the strengths and limitations of this concept in accounting for human fertility variation in both pre- and post-demographic transition populations. Humans fall on the extreme end of already high mammalian parental investment and have highly altricial offspring. It thus seems intuitive that trade-offs between offspring quantity and quality will be fundamental to human life-history evolution [16–18]. Throughout, we treat as uncontroversial the notion that the optimization of the quantity–quality trade-off via natural selection shaped the shared features of our reproductive physiology (i.e. propensity for singleton births, lengthy gestation, lactational amenorrhoea) that ultimately delimit the potential phenotypic range of human fertility. The importance of specific trade-offs nevertheless may vary across different levels (species, populations, and individuals). Accordingly, here we focus our attention on critically evaluating whether, *within the potential phenotypic range of fertility for our species*, the optimization of resource allocation to offspring quantity versus quality can account for observed socio-ecological and individual-level variation in fertility.

We begin by summarizing the contributions of David Lack, a pioneer of life-history theory, to the study of clutch size variation in birds, and the legacy of his research in contemporary studies of animal and human reproductive behaviour. From this platform, we argue that Lack's original focus on clutch size rather than lifetime fertility, in addition to economic demography's focus on the demographic transition rather than variation in pre-demographic transition fertility, has led much of the existing literature to overemphasize the pivotal role of the offspring quantity–quality trade-off in accounting for human fertility variation. This position is supported from a review of the anthropological literature concerning high fertility and high mortality populations somewhat characteristic of our evolutionary past. Here, we argue that a number of important factors reduce costly sibling competition across the life course, so that the relevance of quantity–quality trade-offs to the initiation, continuation, or termination of reproduction is likely to be relatively modest compared with populations that are undergoing or have completed the demographic transition. We then briefly discuss alternative life-history trade-offs that may play a more dominant role in determining the individual variance of fertility in pre-demographic transition environments. To conclude, we revisit the demographic transition and consider how a more holistic appreciation of the multiple life-history trade-offs and selective forces that influence fertility identifies gaps in our current understanding and opportunities for future research.

## 2. The individual optimization of fertility

### (a) Optimizing clutch size

For evolutionary demographers, the idea that contingent trade-offs between offspring quantity and quality determine individual variation in fertility has its roots in the work of the ornithologist David Lack. Lack was among the very first researchers to initiate the application of the ‘Modern Synthesis’, the successful integration of Darwin's theory of natural selection with Mendelian genetics, to the study of animal behaviour [19]. Before Lack, differences in clutch size (i.e. number of offspring born in a single reproductive bout) were understood to be driven by mortality rates with large clutches in high-mortality environments required to perpetuate the *species* [20] (cited in [19, p. 62]). Lack countered that natural selection operates at the *individual level*, so that individuals reproducing at a lower rate than that which maximizes reproductive success are rapidly outcompeted [21]. In his own words:

Clutch size has been evolved through natural selection to correspond with the largest number of young for which the parents can on the average find enough food. [22, p. 32]

This simple hypothesis was one of the first optimization models applied to the study of animal behaviour, contributing substantially to the transformation of ecology from a descriptive discipline to a predictive and experimental science, and earning Lack the epithet ‘father of evolutionary ecology’ [19].

Lack tested his ideas using a novel experimental method, the manipulation of clutch size by transferring newly hatched young to and from nests to create enlarged and reduced broods. Clutch size manipulations have now been conducted on a wide range of avian species where robbing and/or cuckolding parents is a relatively straightforward procedure [10]. The method has also been extended to reptiles [23], insects [24], and even to some mammals where experimentally added pups are not obviously distinguished by breastfeeding mothers [25] or were hormonal treatments have been used to physiologically enlarge litter size [26]. Lack's hypothesis predicts that unmanipulated clutches will produce more surviving young on average than either enlarged or reduced clutches. Some studies report findings largely consistent with the individual optimization of clutch size (e.g. [27]). However, others find that enlarged, or even reduced, clutches produce the most surviving offspring, suggesting that clutch size might be under additional selective pressures. As we explain below, modifications of Lack's initial hypothesis have been suggested to explain such mixed results and to extend this optimality framework to reproductive strategies more generally. Many of these modifications are particularly pertinent to the study of human fertility.

### (b) Extending Lack's model

First, Lack was concerned with the optimization of a single reproductive bout. However, in iteroparous species (i.e. sequential breeders), investment in one clutch will also influence available resources for future survival and reproduction. In other words, considering a single clutch ignores trade-offs between reproduction and somatic maintenance, and consequently between current and future reproduction [28]. This has been used to explain why enlarged clutches often outperform unmanipulated clutches when considering only a single breeding season. For example, in kestrels, enlarged clutches produce more surviving offspring but reduce parental survival and thus opportunities for future reproduction, reducing total fitness across the lifespan ([29], see also [30]). Furthermore, when modelling the evolved ‘decision rules' guiding behaviour, conceptualizing lifetime fertility as equitable to clutch size abstracts from the temporal and sequential aspects of reproductive decision-making. This issue also extends to the work of Becker and much of the subsequent economic literature on fertility, in what Lee describes as the ‘derisive single-litter characterization’ [31, p. 70].

Second, Lack's account implicitly assumes asexual reproduction and uniparental care by neglecting how the dynamics of partner attraction, retention, and coordinated offspring provisioning may influence the resources available for investing in offspring quantity and quality, and the costs and benefits thereof (e.g. [32]). Parental and mating efforts inevitably trade off, albeit in complex species-specific ways [33]. For humans, there is evidence that mating effort in males does not jeopardize their overall fertility, whereas the pattern for females is variable [34]. Later in the manuscript, we elaborate on how trade-offs between mating and parenting efforts may account for variation in human fertility patterns, both before and following the demographic transition. Sexual reproduction also opens up the possibility that sexual conflict over offspring provisioning may alter reproductive strategies in ways unpredicted when assuming no conflict. Selection may favour higher fertility when paternal care is anticipated, but equally initial levels of fertility may alter the pay-offs to parental investment, creating coevolutionary feedback between offspring number and provisioning strategies [32]. Evolved provisioning strategies and decision rules concerning mating dynamics may thus cause substantial variance in fertility by changing the onset and/or continuation of reproduction. Yet, as with the persistent single-litter characterization, it remains common for theoretical models of human fertility variation to assume asexual reproduction—as discussed in Shenk *et al*. [35] and the models reviewed therein.

Third, Lack's evolutionary ecological model identified the optimal clutch size as that of a population assumed to be in equilibrium. This reliance on the assumption that appropriate mechanisms exist which adapt behaviour to the current environment may be unwarranted. Contemporary evolutionary approaches to behaviour incorporate a consideration of the necessarily imperfect mechanisms making up an organism's ‘adaptive toolbox’, providing predictions about when and why we should anticipate departures from strict optimality [36]. Two points are particularly relevant to this review. In reacting to environmental cues, all organisms lack complete and accurate information of the exact fitness consequences of behaviour, which may not be apparent for many generations, and so must rely on imperfect proxies for fitness to select appropriate behavioural alternatives. This brings evolutionary perspectives in line with the notion of ‘bounded rationality’ in behavioural economics [37]. Furthermore, since natural selection adapts behaviour to past not present environments, evolutionary accounts anticipate adaptive lag in the face of environmental change. Although adaptive lag is certainly not unique to humans (see Schlaepfer *et al*. on ‘evolutionary traps’ [38]) it is anticipated to be particularly severe in human populations that have undergone the rapid and dramatic social, economic, and demographic changes accompanying the dawn of agriculture and the industrial revolution [39], and it opens up new possibilities of using culturally transmitted information to afford adaptation [40].

Finally, both life-history theory and economic accounts of fertility, along with related sociological models of family size and achievement [41,42], are united in their shared emphasis on the dilution of parental investment as the dominant factor dictating relationships between offspring quantity and quality. There is evidence that increasing fertility diminishes investment per offspring in both animal (e.g. [43]) and human families (e.g. [42,44]). However, not all parental contributions to offspring quality are subject to dilution, e.g. the social reputation of belonging to a particular family or the quality of genetic inheritance. Moreover, many alternative mechanisms link offspring quantity and quality, the consequences of which may override the importance of investment dilution in certain contexts. Examples of additional costs of high fertility for individual offspring documented in the animal literature include: heightened visibility to predators due to noisier nest sites [43,45], energetic costs of competitive begging [46], consequences of sibling aggression [47], and, for non-dispersing organisms, greater local competition over non-inherited resources in the environment, including mating opportunities [47], effects that bear strong parallels in humans (e.g. [48,49]). Benefits of large sibships that may offset these costs include reduced costs of thermoregulation by huddling nest-mates [50], provisioning from sibling alloparents [51], teaching by older siblings [52], and the ready availability of strategic-alliance partners [53]. Again in humans, such benefits might include co-residential allies in later life [54] and even improved mental health [55]. Ultimately, selection acts on the net product of all of the pathways through which offspring number and well-being are related, thereby shaping reproductive strategies.

## 3. Trade-offs between offspring quantity and quality in humans

Just as studies of clutch size were pioneering for evolutionary studies of animal behaviour, some of the first anthropologists to apply evolutionary models of behaviour, i.e. human behavioural/evolutionary ecologists, focused on variation in fertility [56]. Below, we review evidence for quantity–quality trade-offs prior to the demographic transition across three dimensions: (i) offspring survival, (ii) offspring (embodied, relational, and material) capital, and finally (iii) offspring reproductive success. On the basis of this review, we conclude that, apart from the constraints imposed on reproductive pace by the shared design of human reproductive physiology, there is currently little evidence that limiting fertility to improve offspring quality can be interpreted as a fitness-maximizing reproductive strategy.

### (a) Offspring survival

Available evidence indicates high fertility often compromises child survival in pre-demographic transition settings, but rarely to the extent that reducing fertility can be understood as a strategy to maximize individual lifetime reproductive success (i.e. the total number of surviving offspring). Child survival is under strong selection in human life-history evolution [57]. In high-fertility, high-mortality settings, many offspring die in early childhood (i.e. under 5 years of age), but once adulthood is reached, mortality typically remains low until old age. There is good evidence that tight birth intervals compromise child survival [58]. Blurton Jones [56], for example, demonstrated that tight birth spacing was a strong negative determinant of child survival in !Kung hunter-gatherers. However, studies of the relationship between lifetime fertility and child survival in hunter-gatherer populations have so far failed to find support for the predicted trade-off [59–61], despite the strong constraints of early mortality on fertility in our species [62]. This inconsistency between Blurton Jones's finding of higher mortality after short birth intervals in the !Kung and the failure of subsequent studies to find support for the predicted trade-off, even in the same ethnic group, underscores the point that the costs and benefits of a large sibling set may vary independently of any direct dilution of parental investment, in this case mother's lactation (which typically ceases at the subsequent pregnancy).

In part motivated by this surprising result, a spate of later studies, mostly concerning contemporary small-scale agriculturalist or historical agrarian populations, went on to examine relationships between fertility and child survival. These studies do show that high fertility is associated with low child survival [63–68]. However, only one study to date has observed a downturn in reproductive success (i.e. total number of surviving offspring) at the highest levels of fertility [63]. In all other cases, while the returns to high parity may diminish, more births always are associated with higher reproductive success.

One reason why trade-offs between fertility and child survival may be seemingly absent, or at least insufficiently strong to favour fertility limitation, is the problem of phenotypic correlations [69]. According to this argument, observational studies routinely underestimate trade-offs because they fail to account for relevant differences between individuals. Most obviously, wealthier individuals can afford to invest more in both offspring quantity and quality, masking underlying trade-offs in resource allocation. This issue is well acknowledged in the animal literature, where results of non-experimental studies are deemed ‘unreliable unless a strong case can be made that all relevant variables have been included in the analysis’ [11, p. 149]. Yet, on the other hand, a distinct methodological issue, the misattribution of causality, is likely to result in widespread overestimation of the impact of high fertility and child survival. Indeed, causality may run predominantly in the opposite direction, with high child mortality motivating high fertility. This may occur due to ‘replacement’ or ‘insurance fertility’, whereby parents have additional births to compensate for earlier infant death(s) or expected deaths from causes outside of their control [70,71]. While investigators have attempted to address this issue of reverse causality by excluding very short birth intervals likely to reflect replacing of a dead infant, cut-offs are arbitrary, and there is clearly a need for modelling fertility decisions as a dynamic coevolutionary process both caused by and precipitating child mortality.

There are additional reasons why trade-offs between fertility and child survival may be weak, so that parents do not optimize their fertility decisions on the basis of expected mortality. Child mortality may be high but ‘extrinsic’, i.e. largely care-independent within locally feasible ranges of parental investment, as opposed to ‘intrinsic’, i.e. largely tied to variation in parental investment [59]. This does not mean that children are not highly dependent on parents, but rather that above a readily obtainable threshold, variance within attainable limits of parental care is poorly predictive of mortality [59,72]. Several socio-ecological factors may restrict parental ability to ensure offspring survival, such as unavoidably high pathogen loads, poor sanitation and healthcare access, and vulnerabilities to subsistence failure, natural disasters, and violent conflict. Consistent with this hypothesis, Lawson *et al.* [67], in an analysis of national African demographic surveys, estimate that trade-offs between fertility and child survival are larger in relative magnitude in low-mortality contexts, wherein causes of extrinsic mortality may be reduced (i.e. fewer children die overall but mortality risk is more closely predicted by fertility). Future research is required to explicitly assess the extent to which the dilution of parental care mediates fertility–child mortality relationships across different settings, and more generally to establish the true extent to which child mortality varies along the extrinsic–intrinsic dimension across environments and age ranges.

### (b) Offspring capital among survivors

Evidence that high fertility diminishes offspring capital is mixed in pre-demographic transition populations, with many studies indicating that increasing fertility can be beneficial at even very high levels. With regard to ‘embodied capital’ (i.e. physical and mental well-being, knowledge, skills, etc.), some studies demonstrate that children in larger families have relatively poor physical health, but others find no trade-off or mixed effects depending on sibling characteristics and/or health measures used [73–77]. Data on how the presence of siblings influences mental well-being, knowledge, and skills are scarce in pre-demographic transition contexts. Studies of educational attainment in largely rural contexts in developing countries where fertility remains high have reported both positive and negative associations between sibling number and schooling outcomes [8].

A number of factors may limit costly sibling competition over embodied capital in developing and ‘traditional’ populations. First, while there is probably always a net energetic deficit to rearing children [13], children in rural subsistence economies are typically active producers from an early age. This recruitment of juvenile help partially offsets the dilution of family resources by large sibships [78,79]. Second, it is now clear that alloparenting by wider networks of kin, including grandparents, plays a fundamental role in human life-history evolution. Assistance rearing offspring may buffer the costs of high fertility, particularly when assistance from kin responds flexibility to demand [80,81]. Third, extrinsic environmental risk may extend its influence beyond child mortality to embodied capital among survivors. Substantial variation in offspring outcomes may thus be accounted for by wider socio-ecological conditions beyond parental control, such as environmentally driven fluctuations in food and water insecurity and pathogen load, particularly in the absence of state-level investments in modern healthcare services. Under such conditions, limiting fertility in order to increase parental investment may be somewhat inconsequential with respect to offspring capital. Furthermore, since embodied capital is primarily converted to fitness via survival to reproductive age in traditional relatively high-mortality settings [82], parental perceptions of unavoidable mortality risk (to the extent this is viable [70]) may disincentivize investment more generally, further lowering its relevance as a determinant of offspring success, and thus reducing the impact of sibling competition [83]. We again emphasize that, while such logic is cogent, existing studies of quantity–quality trade-offs in humans have rarely explicitly considered whether parental investment dilution mediates observed relationships between fertility and offspring outcomes. Causal mediation analyses considering alternative forms of parental investment net of the broader set of factors influencing offspring success are lacking. This issue extends to studies evoking resource dilution as the mechanism behind sibship size and education relationships in developed populations [42].

Available evidence suggests ‘relational capital’ (i.e. social ties in food-sharing networks and other forms of assistance) is largely not subject to dilution effects with increasing sibship size. Instead, larger numbers of siblings appear to increase an individual's network of altruists who may provide support across multiple dimensions. Draper & Hames [60], for example, suggest that positive associations between sibship size and fertility in the !Kung are driven by nepotistic aid in the form of food, assistance in childcare, access to foraging territories occupied by dispersed siblings, and political support in times of conflict. More generally, and particularly where there is scope for direct sibling competition, the positive fitness effects of the relational wealth inherent in large families and lineages, as noted in Dominica [84], may be countered by the dilution of material capital among brothers [48,84,85]. Indeed, competition for material wealth or ‘extrasomatic capital’ (i.e. land, livestock, and household goods) is dictated by the extent to which a population holds such resources. Competition over material wealth is relatively limited in forager and simple horticulturalist populations, which lack substantial extra somatic wealth compared to pastoralists and agriculturalists, where land and livestock are essential to local subsistence and are transferred across generations [86]. Resource transfers of this type often occur in tandem with marriage and family formation, and have been studied by anthropologists alongside reproductive outcomes. We therefore summarize the evidence for costly sibling competition over material wealth in the following section.

### (c) Offspring reproductive success

Sibling competition between surviving offspring is only predicted to lead to fertility limitation if it ultimately diminishes offspring reproductive success. To date, trade-offs between offspring number and offspring reproductive success among survivors have only been demonstrated in contexts where material wealth is transmitted across generations, i.e. stemming from the domestication of plants and animals [16]. For example, both Mace [85] and Borgerhoff Mulder [48] demonstrate substantial sibling competition over reproductive success between brothers in east African pastoralist populations where material wealth transfers play a fundamental role in marriage placements; while for daughters (the non-inheriting sex), the existence of additional sisters either had no effect or improved reproductive success [48,85]. Recently, Gibson & Gurmu [87] harnessed a natural experiment to demonstrate the importance of material wealth in establishing sibling competition over reproductive success. They demonstrate that in rural Ethiopian families that underwent a government land redistribution scheme (removing the influence of parental transfers), siblings had little effect on reproductive success. However, for families that were not part of this scheme and where land was inherited, males with more older brothers had smaller farms and lower reproductive success.

Where substantial material wealth is inherited (i.e. in pastoralists and intensive agriculturalists), and where sibling competition potentially reduces reproductive success, formal mathematical models have indicated that the costs of raising and marrying off children could hypothetically favour fertility limitation to maximize fitness [88,89]. However, prior to the demographic transition, it does not seem that the typical solution to these trade-offs has been fertility limitation. Instead, parents have commonly solved this dilemma via differential parental investment. There are various ways, both behavioural and/or institutional, in which this can be achieved. Regarding behavioural strategies, parents can tune their investment in surviving offspring to ensure optimal use of inherited resources, as with favouring inheriting sons [90,91]. Institutional solutions include primogeniture, ultimogeniture, and unigeniture [92–95], conventions on age at first marriage [96], and even complete restrictions on marriage, as among the Kenyan Rendille [97]. Strategies of biased investment, rather than fertility reduction, may be favoured because of significant uncertainty in how many children born will survive to adulthood or have the qualities necessary for the responsibilities of inheritance, with parents following what has been colloquially termed an ‘heirs and spares’ reproductive strategy. Specific models are needed to determine how parents allocate effort between offspring number and investment as a sequential set of decisions contingent on changing conditions (maternal state, child survival, material resources, etc.); without these, anthropologists must continue to speculate on how parents adjust their fertility to their material circumstances. Furthermore, these decisions are rarely in the province of a single individual [89,98], an issue to which we now turn.

## 4. Beyond the quantity–quality trade-off

In the sections above, we have made the argument that, with the exception of avoiding substantial costs of tight birth spacing on infant survival, the costs of high fertility on offspring quality appear largely offset by the benefits of greater offspring quantity, at least in the observed range of our species [62]. In this section, we propose that, in addition to stochastic variation [99,100], fertility variation prior to the demographic transition can be better understood in terms of the individual optimization of alternative life-history trade-offs inherent to iteroparous and sexually reproducing species. Note that in no sense are we refuting the fact that reproductive success is a function of offspring quantity multiplied by offspring quality. Fitness is necessarily measured as the reproductive value of offspring; Fisher [101] recognized that each birth should be weighed by its probability of survival and reproduction, hence the multiplicative term in the reproductive value equation. We also acknowledge that distinguishing life-history trade-offs can analytically be complex since resource allocation decisions have far-reaching and overlapping consequences. However, it is clear that alternative trade-offs can influence resources available for investing in reproduction in ways that cannot be obviously modelled as a quantity–quality trade-off. Accordingly, we now turn to distinct trade-offs that might result in lower fertility than would otherwise be expected by focusing solely on the impacts of fertility on offspring quality. In doing so, we highlight implications with regard to underlying adaptive mechanisms that can account for observed variation in fertility within and between human populations. Primary among these alternative trade-offs are those between reproductive and somatic effort, and between fertility and mating effort. Discussions of these trade-offs are necessarily brief and aimed at highlighting open questions for future research. We also acknowledge that additional trade-offs, beyond the scope of this review, such as that between self- versus nepotistic investment [102], may play a further role in determining individual variation in fertility.

### (a) Trade-offs between reproductive and somatic effort

In the absence of intense competition between offspring for post-natal investment, fertility in pre-demographic transition societies may be best understood as primarily determined by the availability of energetic reserves required for offspring production in pregnancy—redirecting our attention to the life-history trade-off between reproductive and somatic effort. This is particularly true for income breeders [103] where individuals supplement stored energetic reserves with the resources required for reproduction, as in humans who must procure food on a daily basis. Evolutionary anthropologists studying high-fertility settings have demonstrated the fundamental importance of female energetic reserves in curtailing the likelihood of conception and in bringing a conception to term in response to factors such as undernutrition, miscarriage under stress, high workloads, and prolonged breastfeeding [104]. Limiting fertility at such times, so that resources can be allocated to somatic effort, is probably an adaptive mechanism to safeguard against scenarios where current reproduction would jeopardize maternal survival and thus both chances of future reproduction and the survival of existing offspring. These responses appear to be navigated primarily by the adaptive design of female reproductive physiology, with reaction norms based on maternal condition rather than active cognitively engaged monitoring of the potential consequences of current reproduction. It bears noting, however, that women's perceptions of the intersection of their fertility goals and aging bodies is quite developed within some cultural contexts (e.g. [105,106]).

Furthermore, age at first birth in humans reflects a trade-off between the costs of delay (truncating the remaining years available for reproduction), which are elevated in high-mortality environments, with the benefits of postponement via the accruement of personal capital (such as enhanced education and income) [107]. The clearest example of the trade-off between somatic and reproductive effort is the tendency of mothers to delay reproduction until they are fully matured, since reproducing prior to completed adolescent growth jeopardizes maternal growth [108]. Education is another form of somatic investment, with investments in education shown to be associated across many countries with lower fertility [109]. An additional trade-off exists between having another baby and maternal survival, since each birth increases maternal mortality, thereby jeopardizing the survival of existing offspring. This might be construed as a quantity–quality trade-off [64]. The challenge for the empiricist then is to identify the benefits of the survival dividend (earned from the deferred birth): does this dividend lead to additional births later or to increased investment in current offspring? While such subtleties seem difficult to test, techniques that allow analysis of the repetitive decisions a woman makes each year as a function of various changing-state variables can shed light on these dynamics. To take an example from a modern population context, it was long thought that women who pursued education did so at a fertility cost, but recent work based on year-by-year hazard analyses reveals that, in at least some contexts, child bearing impedes education more than the reverse [110]. Given the intricate interdependencies of these posited trade-offs, we suspect that most progress will be made from studies that examine the precise timing of conceptions (or births) as a function of age, of energy budget or income, and of pre-existing offspring (sibling competitors) in the style of Cohen *et al*.'s analysis [110] (see also [111]).

Positioning the trade-off between reproduction and somatic effort as pivotal to reproductive decision-making suggests resource access and maternal energy budgets are fundamental determinants of socio-ecological variation in fertility, rather than the extent to which local ecologies dictate high or low levels of costly sibling competition. As noted above, maternal energy budgets are likely to be particularly pertinent in a species where foraging and/or food acquisition and preparation is a daily concern and can be energetically expensive. A simple prediction might be that better socio-ecological conditions would lead to higher fertility—since more resources are now available for reproduction. However, the situation is complex because better conditions may not only improve maternal energy budgets, but also increase the survival chances of children, thereby increasing the returns to higher investment and altering patterns of replacement and insurance fertility. As we describe below, the demographic transition also clearly bucks the expectation that resource-rich ecologies will lead to greater fertility. More research is required to assess between population variation in human fertility *within pre-demographic transition contexts*, where fertility variation is substantial but still poorly understood from an evolutionary perspective [112,113] and relatively neglected by demographers, who have long been preoccupied with the relatively predictable trends associated with the demographic transition.

### (b) Trade-offs with fertility and mating effort

Despite the common simplifying assumptions of many formal models of fertility in both economics and evolutionary anthropology, human reproduction is a sexual act, thereby entailing the fitness interests of more than one individual. Measures of life-history trade-offs should accordingly incorporate currencies earned through sexual selection. This will produce a more fully unified evolutionary account of fertility that includes social and institutional considerations and constraints [114,115]. For example, there is good reason to believe that mating dynamics play an important role in human fertility variation, in so far as both the age at which individuals enter marriage and the rate at which they change or accumulate mates vary widely across and within populations. The extent to which fertility decisions are shaped by mating strategies has been largely overlooked by evolutionary demographers. However, just as patterns of parental care are increasingly seen as coevolving with interactions within and between the sexes [116,117], so too may fertility decisions.

In a species characterized by relatively stable pair bonds, individuals face a trade-off between starting to reproduce early and waiting to find a preferred mate. Accordingly, variation in age at marriage probably reflects the dynamics involved in mating effort, sensitive to opportunities to get a high-quality mate who can provide both direct and indirect fitness benefits. Such concerns will be particularly relevant when reproduction involves significant resource transfers within the pair bond, again characteristic of human marriage, where males and females typically engage in relatively complementary economic activities [118]. For example, delaying marriage and fertility, thereby truncating the period of life available for future reproduction, may be favoured if time and energy gained can be allocated to enhancing attractiveness and consequently securing (and retaining) a superior mate. There is plenty of evidence that by building up human capital as education a woman can boost her lifetime earnings, thereby increasing the overall budget she can allocate to reproduction [82] and, through educational homogamy, finding an educated spouse (e.g. [119]). However, in rather different social contexts, women may accept very early marriage as a means of securing wealthy and high-ranking husbands, as in 18th to 19th century Germany [120]. In short, strategies to secure high-quality mates may or may not entail delays to reproduction and possibly to reductions in overall fertility, and there is as yet little intersection between theoretical models and empirical variability to guide research in this area.

Turning from first marriage to mate switching, it may pay to slow down reproduction if searching for a replacement mate, and to enhance fertility if aiming to retain a current mate, at least from female point of view. This is because typically remarriage is more difficult for women with children [121] and divorce is more likely in the case of a childless union [122]. With higher variability in mate quality, either sex might be tempted to modulate their fertility to be successful in their optimal mating strategy. This is particularly likely in hunter-gatherer or horticultural populations where in the absence of heritable capital a mate's provisioning abilities can vary quite unpredictably over time due to disease, accident, or other eventualities. More generally, the extent of conflict between spouses [123] is likely to both drive fertility preferences and result from them in ways that have not yet been theorized, in part because of the relatively narrow focus until now on fertility resulting primarily from the resolution of quantity–quality trade-offs.

While the trade-off between reproduction versus some aspects of maternal somatic capital is mainly regulated by physiology, social institutions can play a larger role in determining how mating dynamics influence fertility. For example, if men have the option of polygyny, there may be less pressure on women to produce offspring at a faster rate than is their preference [123]. Similarly, a woman's fertility can be affected by post-marital residence norms, upwards by the presence of her husband's parents, and downwards by the presence of her own parents [98,124]. Women are also more effective in achieving their preferred fertility when their mother lives nearby [125]. In general however, our understanding of these dynamics is largely anecdotal [126]. Furthermore, there is no reason to assume that social institutions regulating marriage and residence are exogenous. People strategize within the culturally agreed-on norms. For example, not all men marry polygynously in a polygynous society, and only some men and women remarry where serial monogamy is the norm, opening up the possibility of a gradual shift in norms in so far as these reflect conformity or frequency dependence. There is accordingly considerable potential for investigating the coevolution of marriage norms with fertility strategies, as pioneered by Goody [94] (see also [127]). For example, Hawkes *et al.* [128] propose that marriage is as a normative solution to a game of coordination among males with respect to reducing the costs of mate guarding. Similarly, flexible norms regarding marriage and divorce in populations where individual capital varies widely over time may emerge because of the high pay-offs to mate switching.

## 5. The demographic transition revisited

As populations undergo socio-economic and cultural ‘modernization’, the factors that once reduced costly sibling competition begin to erode. Children are no longer involved in active resource production and so have little potential to underwrite their own costs to parents. Kin networks become fragmented, and the emergence of low fertility rates leads to a lower absolute number of potential alloparents [129]. Declining extrinsic environmental risks render the returns to parental investment more certain, encouraging greater investment and making the dilution of such investment a greater relative determinant of offspring success. In addition, the scope of sibling competition over material capital is increased dramatically by the introduction of modern skill-based labour economies, where human capital takes longer to instil via formal education and work experience [82,129]. Accordingly, the best evidence of sibling competition in developing populations comes from those that are more economically developed or from relatively urban zones within developing populations [8,16,130]. Indeed, the very fact that most evolutionary anthropological researchers come from such populations may have led to the appeal of the quantity–quality model.

Many evolutionary demographers have argued that modern fertility decline may be adaptive as part of an optimal regulation of the quantity–quality trade-off, provided substantial economic rewards are bestowed on descendants [35,88,131,132]. Multigenerational studies confirm, however, that modern low fertility rates are unlikely to be fitness-maximizing, with low fertility benefiting descendent material and somatic capital, but having little impact on descendant survival or reproductive success [5,6]. In short, parents are not effective in trading off quantity for quality in such a way as to maximize fitness. Rather, parents behave as if ever more investment in offspring will pay off in the competitive market economy, where increasingly rare skills yield increasingly high salaries and social prestige [82]. Furthermore, they appear motivated to imitate the investment patterns of the most prestigious members of the community [133,134], with diverse values generating increasingly complex cultural evolutionary dynamics [40,135]. However, despite the fact that families are patently much smaller than any optimizing model would predict, there is a sense in which the quantity–quality trade-off holds—at least through the *perception* of quality. This suggests there is adaptive lag in mechanisms governing fertility. Under modernization, humans elevate their perception of the costs of high fertility on offspring capital, leading to a correspondingly exaggerated strategy of fertility limitation [16,35,136]. For example, echoing Lack's clutch size hypothesis, Kaplan *et al*. propose that that:

Because human parents and grandparents provision children, natural selection probably produced mechanisms by which fertility could respond to the number of children parents could afford to raise in any given socio-ecology. [136, p. 238]

In modern environments such adaptive mechanisms then lead to maladaptively low fertility in response to increased perceived costs of child rearing well outside the range experienced previously by our ancestors.

On the basis of the literature reviewed across previous sections, we highlight two important considerations stemming from a more holistic appreciation of the multiple selective forces acting on human-fertility variation. First, if it is indeed true that modern fertility limitation is best understood as a strategy to advance offspring status in line with a perceived quantity–quality trade-off, then this represents a potentially radical shift. As we have argued, there is little indication that fertility variation can be accounted for by the tactical balancing of equivalent forms of this trade-off in pre-demographic transition environments. In this sense, limiting fertility to strategically enhance offspring success cannot be considered a straightforward extension of pre-existing reproductive strategies. This raises very interesting questions about the flexibility of human behaviour under rapidly changing environments, and identifies the need for a more developed study of the cognitive mechanisms underlying human reproduction to match the substantial progress made in our understanding of the physiology of human reproduction across the 1980s and 1990s [104]. Clearly, a sharper understanding of the mechanisms contributing to fertility variability in pre-demographic transition populations lacking modern birth control is critical to any empirical anchoring of such speculations. Future work would also be usefully concentrated on those populations undergoing modernization and engaging with such technologies for the first time (e.g. [137]).

Second, we caution that while data on the apparent benefits of low fertility to descendant material capital certainty appear consistent with the view that perceived quantity–quality trade-offs drive the demographic transition, evolutionary anthropologists' persistent preoccupation with this trade-off means that relatively little attention has been paid to the possibility that alternative trade-offs play a substantial role in modern fertility patterns. Indeed, it remains an open possibility that modern low fertility may be better understood in terms of exaggerated returns of low fertility to own socio-economic success relatively independently of the socio-economic consequences of costly sibling competition. Accordingly, we should perhaps refocus our attention more on the factors that lead individuals to postpone fertility, i.e. the decision to reproduce now versus later, rather than the decision of how many offspring to have [138]. This perspective has the obvious advantage of accounting for why many people accrue capital at the expense of having no kids at all. There is also much scope for improving our understanding of how novel features of modern mating markets might lead to low fertility. For example, high-density mating markets may lead to extreme investments in choosiness, and such long search times, lengthy courtship, and possibly higher rates of partner switching in response to the perception that a potentially better mate may always be available [139]. Such modern mating markets may indirectly reduce fertility by reducing the amount of time individuals spend in reproductively viable partnerships. As Moya *et al*. [126] review, there is also a need for greater attention to the possibility that sexual conflict may influence fertility optima in certain contexts.

## 6. Conclusion

The optimization of the life-history trade-off between offspring quantity and quality is surely fundamental in defining the theoretical upper limits of human fertility and our propensity for singleton births. Yet the extent to which it can meaningfully account for why so few women approach maximal fertility and for the substantial ecological and individual variance in fertility rates observed even before the demographic transition remains an open question. Our review of sibling competition in high-fertility, high-mortality populations leads us to propose that alternative life-history trade-offs, such as that between reproductive and somatic effort, and between fertility and mating effort, play a more pivotal role in accounting for fertility variation in settings characteristic of our evolutionary past. Even where high fertility has notable costs on offspring reproductive success due to substantial material wealth transfers at marriage and inheritance, a relatively novel form of sibling competition following the domestication of plants and animals, this dilemma has typically been solved by biased parental investment rather than tactical fertility reduction.

The trade-off between the perceived quality and quantity of offspring is more obviously relevant to decisions to reduce fertility accompanying socio-economic and cultural modernization, an observation that may account for our preoccupation with this trade-off, since researchers themselves are living in environments where the direct and opportunity costs of raising children are particularly salient. However, even in modern low-fertility contexts, we suggest that expanding present theoretical frameworks beyond the persistent yet artificial single clutch and asexual reproduction assumptions, dating back to both Lack and Becker, will be necessary to account for observed fertility patterns. The quantity–quality trade-off concept has propelled a vast literature addressing species, population, and individual-level variation in reproductive strategies. Nevertheless, we will ultimately understand a lot more about reproductive decision-making by enriching current frameworks with greater attention to weighing up the relative contribution of the quantity–quality trade-off to the alternative selective forces discussed in this paper.

## References

[1] Doepke M 2015 Gary Becker on the quantity and quality of children. J. Demogr. Econ. 81, 59–66. (10.1017/dem.2014.8)

[2] Becker GS 1960 An economic analysis of fertility. Demographic and economic change in developed countries. Princeton, NJ: Princeton University Press.

[3] Becker GS, Lewis HG 1973 On the interaction between quantity and quality of children. J. Polit. Econ. 81, S279–S288. (10.1086/260166)

[4] Lam D 2003 Evolutionary biology and rational choice in models of fertility. In Offspring: human fertility behaviour in biodemographic perspective (eds Wachter KW, Bulatao RA), pp. 322–338. Washington, DC: National Academies Press. 22675746

[5] Goodman A, Koupil I, Lawson DW 2012 Low fertility increases descendant socioeconomic position but reduces long-term fitness in a modern post-industrial society. Proc. R. Soc. B 279, 4342–4351. (10.1098/rspb.2012.1415) PMC3479798 22933371

[6] Kaplan HS, Lancaster JB, Johnson SE, Bock JA 1995 Does observed fertility maximize fitness among New Mexican men? A test of an optimality model and a new theory of parental investment in the embodied capital of offspring. Hum. Nat. 6, 325–360. (10.1007/BF02734205) 24203123

[7] Van Bavel J, Moreels S, Van de Putte B, Matthijs K 2011 Family size and intergenerational social mobility during the fertility transition: evidence of resource dilution from the city of Antwerp in nineteenth century Belgium. Demogr. Res. 24, 313–344. (10.4054/DemRes.2011.24.14)

[8] Eloundou-Enyegue PM, Williams L 2006 Family size and schooling in sub-Saharan African settings: a reexamination. Demography 43, 25–52. (10.1353/dem.2006.0002) 16579207

[9] Lawson DW 2011 Life history theory and human reproductive behaviour. In Evolutionary psychology: a critical introduction (ed. Swami V), pp. 183–214. Oxford, UK: BPS Blackwell.

[10] Stearns SC 1992 The evolution of life histories. Oxford, UK: Oxford University Press.

[11] Roff DA 2002 Life history evolution. Sunderland, MA: Sinauer Associates.

[12] Robson AJ 2001 The biological basis of economic behavior. J. Econ. Lit. 39, 11–33. (10.1257/jel.39.1.11)

[13] Kaplan H 1994 Evolutionary and wealth flows theories of fertility: empirical tests and new models. Popul. Dev. Rev. 20, 753–791. (10.2307/2137661)

[14] Kaplan HS, Bock JA, Hooper PL 2015 Fertility theory: embodied-capital theory of life history evolution. International encyclopedia of the social & behavioral sciences, pp. 28–34, 2nd edn Oxford, UK: Elsevier.

[15] Wachter KA 2003 Biodemography of fertility and family formation. In Offspring: human fertility behaviour in biodemographic perspective (eds Wachter KW, Bulatao RA), pp. 1–17. Washington, DC: National Academies Press. 22675746

[16] Lawson DW, Mace R 2011 Parental investment and the optimization of human family size. Phil. Trans. R. Soc. B 366, 333–343. (10.1098/rstb.2010.0297) 21199838 PMC3013477

[17] Borgerhoff Mulder M 2000 Optimizing offspring: the quantity–quality tradeoff in agropastoral Kipsigis. Evol. Hum. Behav. 21, 391–410. (10.1016/S1090-5138(00)00054-4) 11146305

[18] Walker RS, Gurven M, Burger O, Hamilton MJ 2008 The trade-off between number and size of offspring in humans and other primates. Proc. R. Soc. B 275, 827–834. (10.1098/rspb.2007.1511) PMC2596903 18077252

[19] Anderson T 2013 The life of David Lack: father of evolutionary ecology. Oxford, UK: Oxford University Press.

[20] Pettingill OS 1946 A laboratory and field manual of ornithology. Minneapolis, MN: Burges Publishing Co.

[21] Lack D 1947 The significance of clutch-size. Ibis 89, 302–352. (10.1111/j.1474-919X.1947.tb04155.x)

[22] Lack D 1954 The natural regulation of animal numbers. Oxford, UK: Clarendon Press.

[23] Aubret F, Bonnet X, Shine R, Maumelat S 2003 Clutch size manipulation, hatching success and offspring phenotype in the ball python (*Python regius*). Biol. J. Linn. Soc. 78, 263–272. (10.1046/j.1095-8312.2003.00169.x)

[24] Hardy ICW, Griffiths NT, Godfray HCJ 1992 Clutch size in a parasitoid wasp: a manipulation experiment. J. Anim. Ecol. 61, 121–129. (10.2307/5515)

[25] Koivula M, Koskela E, Mappes T, Oksanen TA 2003 Cost of reproduction in the wild: manipulation of reproductive effort in the bank vole. Ecology 84, 398–405. (10.1890/0012-9658(2003)084%5B0398:CORITW%5D2.0.CO;2)

[26] Oksanen TA, Koskela E, Mappes T 2002 Hormonal manipulation of offspring number: maternal effort and reproductive costs. Evolution 56, 1530–1537. (10.1111/j.0014-3820.2002.tb01463.x) 12206251

[27] Pettifor RA, Perrins CM, McCleery RH 2008 The individual optimization of fitness: variation in reproductive output, including clutch size, mean nestling mass and offspring recruitment, in manipulated broods of great tits *Parus major*. J. Anim. Ecol. 70, 62–79. (10.1111/j.1365-2656.2001.00465.x)

[28] Williams GC 1966 Natural selection, the costs of reproduction, and a refinement of Lack's principle. Am. Nat. 100, 687–690. (10.1086/282461)

[29] Dijkstra C, Bult A, Biflsma S, Dann S, Meiger T, Zijlstra M 1990 Brood size manipulations in the kestrel (*Falco tinnunculus*): effects on offspring and parent survival. J. Anim. Ecol. 59, 269–285. (10.2307/5172)

[30] Gustafsson L, Sutherland WJ 1988 The costs of reproduction in the collared flycatcher *Ficedula albicollis*. Nature 335, 813–815. (10.1038/335813a0)

[31] Lee R 2015 Becker and the demographic transition. J. Demogr. Econ. 81, 67–74. (10.1017/dem.2014.9) PMC4547387 26316929

[32] Smith HG, Härdling R 2000 Clutch size evolution under sexual conflict enhances the stability of mating systems. Proc. R. Soc. B 267, 2163–2170. (10.1098/rspb.2000.1264)

[33] Stiver KA, Alonzo SH 2009 Parental and mating effort: is there neccessarily a trade-off? Int. J. Behav. Biol. 115, 1101–1126. (10.1111/j.1439-0310.2009.01707.x)

[34] Brown GR, Laland KN, Borgerhoff Mulder M 2009 Bateman's principles and human sex roles. Trends Ecol. Evol. 24, 297–304. (10.1016/j.tree.2009.02.005) 19403194 PMC3096780

[35] Shenk MK, Kaplan HS, Hooper PL 2016 Status competition, inequality, and fertility: implications for the demographic transition. Phil. Trans. R. Soc. B 371, 20150150 (10.1098/rstb.2015.0150) 27022077 PMC4822430

[36] Patridge L, Sibly R, Beverton RJH, Hill WG 1991 Constraints on the evolution of life histories and discussion. Phil. Trans. R. Soc. B 332, 3–13. (10.1098/rstb.1991.0027)

[37] Gigerenzer G, Selten R 2002 Rethinking rationality. In Bounded rationality: the adaptive toolbox (eds Gigerenzer G, Selten R), pp. 1–12. Oxford, UK: Oxford University Press.

[38] Schlaepfer MA, Runge MC, Sherman PW 2002 Ecological and evolutionary traps. Trends Ecol Evol. 17, 474–480. (10.1016/S0169-5347(02)02580-6)

[39] Irons W 1998 Adaptively relevant environments versus the environment of evolutionary adaptedness. Evol. Anthropol. Issues News Rev. 6, 194–204. (10.1002/(SICI)1520-6505(1998)6:6<194::AID-EVAN2>3.0.CO;2-B)

[40] Colleran H 2016 The cultural evolution of fertility decline. Phil. Trans. R. Soc. B 371, 20150152 (10.1098/rstb.2015.0152) 27022079 PMC4822432

[41] Blake J 1989 Family size and achievement. Berkeley, CA: University of California Press.

[42] Downey DB 2000 Number of siblings and intellectual development: the resource dilution explanation. Am. Psychol. 56, 497–504. (10.1037/0003-066X.56.6-7.497) 11413873

[43] Lima S 1987 Clutch size in birds: a predation perspective. Ecology 68, 1062–1070.

[44] Lawson DW, Mace R 2009 Trade-offs in modern parenting: a longitudinal study of sibling competition for parental care. Evol. Hum. Behav. 30, 170–183. (10.1016/j.evolhumbehav.2008.12.001)

[45] Skutch AF 1949 Do tropical birds rear as many offspring as they can nourish? Ibis 91, 430–458. (10.1111/j.1474-919X.1949.tb02293.x)

[46] Harper AB 1986 The evolution of begging: sibling competition and parent–offspring conflict. Am. Nat. 128, 99–114. (10.1086/284542)

[47] Mock D, Parker GA 1997 The evolution of sibling rivarily. New York, NY: Oxford University Press.

[48] Borgerhoff Mulder M 1998 Brothers and sisters: how sibling interactions affect optimal parental allocations. Hum. Nat. 9, 119–162. (10.1007/s12110-998-1001-6) 26197443

[49] Macfarlan SJ, Walker RS, Flinn MV, Chagnon NA 2014 Lethal coalitionary aggression and long-term alliance formation among Yanomamö men. Proc. Natl Acad. Sci. USA 111, 16 662–16 669. (10.1073/pnas.1418639111) PMC4250129 25349394

[50] Parker GA, Mock DW 1986 Advantages and disadvantages of egret and heron brood reduction. Evolution 40, 459–470. (10.2307/2408569) 28556322

[51] Malcom J, Marten K 1982 Natural selection and the communal rearing of pups in African wild dogs (*Lycaon pictus*). Behav. Ecol. Sociobiol. 10, 1–13. (10.1007/BF00296390)

[52] Edwards TC Jr 1989 Similarity in the development of foraging mechanics among sibling ospreys. Condor 1, 30–36. (10.2307/1368145)

[53] Lee P 1987 Sibships: Cooperation and competition among immature vervet monkeys. Primates 28, 47–59. (10.1007/BF02382182)

[54] Hill KR et al. 2011 Co-residence patterns in hunter-gatherer societies show unique human social structure. Science 331, 1286–1289. (10.1126/science.1199071) 21393537

[55] Lawson DW, Mace R 2010 Siblings and childhood mental health: evidence for a later-born advantage. Soc. Sci. Med. 70, 2061–2069. (10.1016/j.socscimed.2010.03.009) 20381935

[56] Blurton Jones N 1986 Bushman birth spacing: a test for optimal interbirth intervals. Ethol. Sociobiol. 7, 91–105. (10.1016/0162-3095(86)90002-6)

[57] Jones JH 2009 The force of selection on the human life cycle. Evol. Hum. Behav. 30, 305–314. (10.1016/j.evolhumbehav.2009.01.005) 22003281 PMC3193054

[58] Hobcraft J, McDonald JW, Rutstein S 1983 Child-spacing effects on infant and early child mortality. Popul. Index 49, 585–618. (10.2307/2737284)

[59] Pennington R, Harpending H 1988 Fitness and fertility among kalahari !Kung. Am. J. Phys. Anthropol. 77, 303–319. (10.1002/ajpa.1330770304) 3228170

[60] Draper P, Hames R 2000 Birth order, sibling investment, and fertility among Ju/’Hoansi (!Kung). Hum. Nat. 11, 117–156. (10.1007/s12110-000-1016-0) 26193364

[61] Hill K, Hurtado AM 1996 Ache life history: the ecology and demography of a foraging people. New York, NY: Aldine de Gruyter.

[62] Jones JH, Tuljapurkar S 2015 Measuring selective constraint on fertility in human life histories. Proc. Natl Acad. Sci. USA 112, 8982–8986. (10.1073/pnas.1422037112) 26150499 PMC4517261

[63] Strassmann BI, Gillespie B 2002 Life-history theory, fertility and reproductive success in humans. Proc. R. Soc. B 269, 553–562. (10.1098/rspb.2001.1912) PMC1690939 11916470

[64] Penn DJ, Smith KR 2007 Differential fitness costs of reproduction between the sexes. Proc. Natl Acad. Sci. USA 104, 553–558. (10.1073/pnas.0609301103) 17192400 PMC1766423

[65] Gillespie DOS, Russell AF, Lummaa V 2008 When fecundity does not equal fitness: evidence of an offspring quantity versus quality trade-off in pre-industrial humans. Proc. R. Soc. B 275, 713–722. (10.1098/rspb.2007.1000) PMC2366115 18211874

[66] Meij JJ, van Bodegom D, Ziem JB, Amankwa J, Polderman AM, Kirkwood TBL, De Craen AJM, Zwaan BJ, Westerndorp RGJ 2009 Quality–quantity trade-off of human offspring under adverse environmental conditions. J. Evol. Biol. 22, 1014–1023. (10.1111/j.1420-9101.2009.01713.x) 19298492

[67] Lawson DW, Alvergne A, Gibson MA 2012 The life-history trade-off between fertility and child survival. Proc. R. Soc. B 279, 4755–4764. (10.1098/rspb.2012.1635) PMC3497086 23034700

[68] Stevenson JC, Everson PM, Grimes M 2004 Reproductive measures, fitness, and migrating Mennonites: an evolutionary analysis. Hum. Biol. 76, 667–687. (10.1353/hub.2005.0010) 15757240

[69] van Noordwijk A, de Jong G 1986 Acquisition and allocation of resources: their influence on variation in life history tactics. Am. Nat. 128, 137–142. (10.1086/284547)

[70] Montgomery MR 2000 Perceiving mortality decline. Popul. Dev. Rev. 26, 795–819. (10.1111/j.1728-4457.2000.00795.x)

[71] Randall S, Legrand T 2003 Reproductive strategies and decisions in Senegal: the role of child mortality. Population-E 58, 687–715.

[72] Quinlan RJ 2007 Human parental effort and environmental risk. Proc. R. Soc. B 274, 121–125. (10.1098/rspb.2006.3690) PMC1679876 17134996

[73] Hagen EH, Hames RB, Craig NM, Lauer MT, Price ME 2001 Parental investment and child health in a Yanomamö village suffering short-term food stress. J. Biosoc. Sci. 33, 503–528. (10.1017/S002193200100503X) 11683222

[74] Hagen EH, Barrett HC, Price ME 2006 Do human parents face a quantity-quality tradeoff?: evidence from a Shuar community. Am. J. Phys. Anthropol. 130, 405–418. (10.1002/ajpa.20272) 16365856

[75] Strassmann BI 2011 Cooperation and competition in a cliff-dwelling people. Proc .Natl Acad. Sci. USA 108, 10 894–10 901. (10.1073/pnas.1100306108) PMC3131812 21690343

[76] Hadley C, Belachew T, Lindstrom D, Tessema F 2011 The shape of things to come? household dependency ratio and adolescent nutritional status in rural and urban Ethiopia. Am. J. Phys. Anthropol. 144, 643–652. (10.1002/ajpa.21463) 21404240 PMC3907944

[77] Helfrecht C, Meehan CL In press Sibling effects on nutritional status: Intersections of cooperation and competition across development. Am. J. Hum. Biol. (10.1002/ajhb.22763) 26179564

[78] Kramer KL 2005 Children's help and the pace of reproduction: cooperative breeding in humans. Evol. Anthropol. Issues News Rev. 14, 224–237. (10.1002/evan.20082)

[79] Lee RD, Kramer KL 2004 Children's economic roles in the Maya family life cycle: Cain, Caldwell and, Chayanov revisited. Popul. Dev. Rev. 28, 475–499. (10.1111/j.1728-4457.2002.00475.x)

[80] Sear R, Mace R 2008 Who keeps children alive? A review of the effects of kin on child survival. Evol. Hum. Behav. 29, 1–18. (10.1016/j.evolhumbehav.2007.10.001)

[81] Hrdy SB 2009 Mothers and others. Cambridge, MA: Belknap Press.

[82] Kaplan H 1996 A theory of fertility and parental investment in traditional and modern human societies. Am. J. Phys. Anthropol. 101, 91–135. (10.1002/(SICI)1096-8644(1996)23+<91::AID-AJPA4>3.0.CO;2-C)

[83] Gibson MA, Lawson DW 2011 ‘Modernization’ increases parental investment and sibling resource competition: evidence from a rural development initiative in Ethiopia. Evol. Hum. Behav. 32, 97–105. (10.1016/j.evolhumbehav.2010.10.002)

[84] Quinlan RJ, Flinn MV 2005 Kinship and reproduction in a Caribbean community. Hum. Nat. 16, 32–57. (10.1007/s12110-005-1006-3) 26189515

[85] Mace R 1996 Biased parental investment and reproductive success in Gabbra pastoralists. Behav. Ecol. Sociobiol. 38, 75–81. (10.1007/s002650050219) 12292074

[86] Borgerhoff Mulder M et al. 2009 Intergenerational wealth transmission and the dynamics of inequality in small-scale societies. Science 326, 682–688. (10.1126/science.1178336) 19900925 PMC2792081

[87] Gibson MA, Gurmu E 2011 Land inheritance establishes sibling competition for marriage and reproduction in rural Ethiopia. Proc. Natl Acad. Sci. USA 108, 2200–2204. (10.1073/pnas.1010241108) 21262826 PMC3038728

[88] Mace R 1998 The coevolution of human fertility and wealth inheritance strategies. Phil. Trans. R. Soc. B 353, 389–397. (10.1098/rstb.1998.0217) 9569432 PMC1692221

[89] Luttbeg B, Borgerhoff Mulder M, Mangel M 2000 To marry again or not? A dynamic model for demographic transition. In Human behavior and adaptation: an anthropological perspective (eds Cronk L, Chagnon NA, Irons W), pp. 345–368. New York, NY: Aldine de Gruyter.

[90] Voland E 1988 Differential infant and child mortality in evolutionary perspective: DAta from late 17th to 19th century Ostfriesland (Germany). In Human reproductive behaviour: a Darwinian perspective (eds Betzig L, Borgerhoff Mudler M), pp. 253–261. Cambridge, UK: Cambridge University Press.

[91] Das Gupta M, Zhenghua J, Bohua L, Zhenming X, Chung W, Hwa-OK B 2003 Why is son preference so persistent in East and South Asia? A cross-country study of China, India and the Republic of Korea. J. Dev. Stud. 40, 153–187. (10.1080/00220380412331293807)

[92] Strassmann BI, Clarke A 1998 Ecological constraints on marriage in rural Ireland. Evol. Hum. Behav. 19, 33–35. (10.1016/S1090-5138(97)00103-7)

[93] Hrdy SB, Judge DS 1993 Darwin and the puzzle of primogeniture. Hum. Nat. 4, 1–45. (10.1007/BF02734088) 24214292

[94] Goody J 1976 Production and reproduction: a comparative study of the domestic doman. Cambridge, NY: Cambridge University Press.

[95] Beise J, Voland E 2008 Intrafamilial resource competition and mate competition shaped social-group-specific natal disperal in the 18th and 19th century Krummhörn population. Am. J. Hum. Biol. 20, 325–336. (10.1002/ajhb.20730) 18186514

[96] Hajnal J 1965 European marriage patterns in perspective. In Population in history (eds Glass D, Eversley D), pp. 101–143. London, UK: Edward Arnold.

[97] Roth EA 1993 Re-examination of Rendille population regulation. Am. Anthropol. 95, 597–612. (10.1525/aa.1993.95.3.02a00030)

[98] Leonetti DL, Nath DC, Hemam NS 2007 In-law conflict: women's reproductive lives and the roles of their mothers and husbands among the matrilineal Khasi. Curr. Anthropol. 48, 861–890. (10.1086/520976)

[99] Hruschka DJ, Burger O 2016 How does variance in fertility change over the demographic transition? Phil. Trans. R. Soc. B 371, 20150155 (doi:1098/rstb.2015.0155) 27022082 10.1098/rstb.2015.0155 PMC4822435

[100] Winterhalder B, Leslie P 2002 Risk-sensitive fertility: the variance compensation hypothesis. Evol. Hum.Behav. 23, 59–82. (10.1016/S1090-5138(01)00089-7)

[101] Fisher RA 1930 The genetical theory of natural selection: a complete variorum edition. Oxford, UK: Oxford University Press.

[102] Voland E 1998 Evolutionary ecology of human reproduction. Annu. Rev. Anthropol. 27, 347–374. (10.1146/annurev.anthro.27.1.347) 12295433

[103] Houston AI, Stephens PA, Boyd IL, Harding KC, McNamara JM 2007 Capital or income breeding? A theoretical model of female reproductie strategies. Behav. Ecol. 18, 241–250. (10.1093/beheco/arl080)

[104] Vitzthum V 2009 The ecology and evolutionary endocrinology of reproduction in the human female. Am. J. Phys. Anthropol. 49, 95–136. (10.1002/ajpa.21195) 19890865

[105] Johnson-Hanks J 2002 On the limits of the life cycle in ethnography: towards a theory of vital conjunctures. Am. Anthropol. 104, 865–880. (10.1525/aa.2002.104.3.865)

[106] Mace R 2000 Evolutionary ecology of human life history. Anim. Behav. 59, 1–10. (10.1006/anbe.1999.1287) 10640361

[107] Nettle D 2011 Flexibility in reproductive timing in human females: integrating ultimate and proximate explanations. Phil. Trans. R. Soc. B 366, 357–365. (10.1098/rstb.2010.0073) 21199840 PMC3013465

[108] Rah JH, Christian P, Shamim AA, Arju UT, Labrique AB, Rashid M 2008 Pregnancy and lactation hinder growth and nutritional status of adolescent girls in rural Bangladesh. J. Nutrtition 138, 1505–1511. 10.1093/jn/138.8.1505 18641198

[109] Castro Martín T 1995 Women's education and fertility: results from 26 demograhic and health surveys. Stud. Fam. Plann. 26, 187–202. (10.2307/2137845) 7482677

[110] Cohen JE, Kravdal Ø, Keilman N 2011 Childbearing impeded education more than education impeded childbearing among Norwegian women. Proc. Natl Acad. Sci. USA 108, 11 830–11 835. (10.1073/pnas.1107993108) PMC3141966 21730138

[111] Stulp G, Barrett L 2016 Wealth, fertility and adaptive behaviour in industrial populations. Phil. Trans. R. Soc. B 371, 20150153 (10.1098/rstb.2015.0153) 27022080 PMC4822433

[112] Sellen DW, Mace R 1997 Fertility and mode of subsistence: a phylogenetic analysis. Curr. Anthropol. 38, 878–889. (10.1086/204677)

[113] Bentley GR, Goldberg T, Jasieńska GZY 1993 The fertility of agricultural and non-agricultural traditional societies. Popul. Stud. 47, 269–281. (10.1080/0032472031000147006)

[114] Kaplan HS, Lancaster JB 2003 An evolutionary and ecological analysis of human fertility, mating patterns, and parental investment. In Offspring: human fertility behaviour in biodemographic perspective (eds Bulatao R, Wachter KW), pp. 170–223. Washington, DC: National Academies Press. 22675746

[115] Ross C et al. In press Evidence for quantity-quality trade-offs, sex-specific parental investment, and variance compensation in colonized Agta foragers undergoing demographic transition. Evol. Hum. Behav.

[116] Alonzo SH, Klug H 2012 Maternity, paternity and parental care. In The evolution of parental care (eds Royle NJ, Smiseth PT, Kölliker M), pp. 189–205. Oxford, UK: Oxford University Press.

[117] Kokko H, Jennions MD 2008 Parental investment, sexual selection and sex ratios. J. Evol. Biol. 21, 919–948. (10.1111/j.1420-9101.2008.01540.x) 18462318

[118] Kaplan HS, Hooper PL, Gurven M 2009 The evolutionary and ecological roots of human social organization. Phil. Trans. R. Soc. B 364, 3289–3299. (10.1098/rstb.2009.0115) 19805435 PMC2781874

[119] Borgerhoff Mulder M 1995 Bridewealth and its correlates: quantifying changes over time. Curr. Anthropol. 36, 573–603. (10.1086/204405)

[120] Voland E, Engel MC 1990 Female choice in humans: a conditional mate selection strategy of the Krummhörn women (Germany, 1720–1874). Ethology 84, 144–154. (10.1111/j.1439-0310.1990.tb00791.x)

[121] Buckle L, Gallup GG Jr, Rodd ZA 1996 Marriage as a reproductive contract: patterns of marriage, divorce, and remarriage. Ethol. Sociobiol. 17, 363–377. (10.1016/S0162-3095(96)00075-1)

[122] Betzig L 1989 Causes of conjual dissolution: a cross-cultural study. Curr. Anthropol. 30, 654–676. (10.1086/203798)

[123] Borgerhoff Mulder M, Rauch KL 2009 Sexual conflict in humans: variations and solutions. Evol. Anthropol. Issues News Rev. 18, 201–214. (10.1002/evan.20226)

[124] Sear R, Mace R, McGregor I 2003 The effects of kin on female fertility in rural Gambia. Evol. Hum. Behav. 24, 25–42. (10.1016/S1090-5138(02)00105-8)

[125] Borgerhoff Mulder M 2009 Tradeoffs and sexual conflict over women's fertility preferences in Mpimbwe. Am. J. Hum. Biol. 21, 478–487. (10.1002/ajhb.20885) 19263412

[126] Moya C, Snopkowski K, Sear R 2016 What do men want? Re-examining whether men benefit from higher fertility than is optimal for women. Phil. Trans. R. Soc. B 371, 20150149 (10.1098/rstb.2015.0149) 27022076 PMC4822429

[127] Fortunato L, Archetti M 2010 Evolution of monogamous marriage by maximization of inclusive fitness. J. Evol. Biol. 23, 149–156. (10.1111/j.1420-9101.2009.01884.x) 20069721

[128] Hawkes K, Rogers AR, Charnov E 1995 The male's dilemma: increased offspring production is more paternity to steal. Evol. Ecol. 9, 662–677. (10.1007/BF01237661)

[129] Turke PW 1989 Evolution and the demand for children. Popul. Dev. Rev. 15, 61–90. (10.2307/1973405)

[130] Desai S 1995 When are children from large families disadvantaged? Evidence from cross-national analyses. Popul. Stud. 49, 195–210. (10.1080/0032472031000148466)

[131] Boone JL, Kessler KL 1999 More status or more children? Social status, fertility reduction, and long-term fitness. Evol. Hum. Behav. 20, 257–277. (10.1016/S1090-5138(99)00011-2)

[132] Hill SE, Reeve HK 2005 Low fertility in humans as the evolutionary outcome of snowballing resource games. Behav. Ecol. 16, 398–402. (10.1093/beheco/ari001)

[133] Li NP, Lim AJY, Tsai MH, Jiaqing O 2015 Too materialistic to get married and have children? PLoS ONE 10, e0126543 (10.1371/journal.pone.0126543) 25955502 PMC4425653

[134] Richerson PJ, Boyd R 2008 Not by genes alone: how culture transformed human evolution. Chicago, IL: University of Chicago Press.

[135] Kolk M, Cownden D, Enquist M 2014 Correlations in fertility across generations: can low fertility persist? Proc. R. Soc. B 281, 20132561 (10.1098/rspb.2013.2561) PMC3924067 24478294

[136] Kaplan H, Lancaster JB, Tucker WT, Anderson KG 2002 Evolutionary approach to below replacement fertility. Am. J. Hum. Biol. 14, 233–256. (10.1002/ajhb.10041) 11891936

[137] Alvergne A, Lawson DW, Clarke PMR, Gurmu E, Mace R 2013 Fertility, parental investment, and the early adoption of modern contraception in rural Ethiopia. Am. J. Hum. Biol. 25, 107–115. (10.1002/ajhb.22348) 23180659

[138] Buckles K 2008 Understanding the returns to delayed childbearing for working women. Am. Econ. Rev. 98, 403–407. (10.1257/aer.98.2.403)

[139] Kokko H, Rankin DJ 2006 Lonely hearts or sex in the city? Density-dependent effects in mating systems. Phil. Trans. R. Soc. B 28, 319–334. (10.1098/rstb.2005.1784) PMC1569612 16612890

